# Pegylated Liposomal Doxorubicin in Vindesine-Based and Bortezomib-Based Regimens for Patients With Newly Diagnosed Multiple Myeloma: A Retrospective Study of Efficacy and Safety

**DOI:** 10.3389/fonc.2021.597453

**Published:** 2021-03-25

**Authors:** Yujia Zhai, Dai Yuan, Xueling Ge, Shunfeng Hu, Peipei Li, Xiaosheng Fang, Ying Li, Xiangxiang Zhou, Xin Wang

**Affiliations:** ^1^ Department of Hematology, Shandong Provincial Hospital, Cheeloo College of Medicine, Shandong University, Jinan, China; ^2^ School of Medicine, Shandong University, Jinan, China; ^3^ Department of Hematology, Shandong Provincial Hospital Affiliated to Shandong First Medical University, Jinan, China; ^4^ Shandong Provincial Engineering Research Center of Lymphoma, Jinan, China; ^5^ Branch of National Clinical Research Center for Hematologic Diseases, Jinan, China; ^6^ National Clinical Research Center for Hematologic Diseases, The First Affiliated Hospital of Soochow University, Suzhou, China

**Keywords:** pegylated liposomal doxorubicin, multiple myeloma, efficacy, survival, toxicity

## Abstract

**Purpose:**

Although pegylated liposomal doxorubicin (PLD) has been approved in combination with bortezomib for relapsed/refractory multiple myeloma (MM), the antitumor efficacy and tolerability of PLD in different regimens for patients with newly diagnosed MM (NDMM) have not been fully defined.

**Methods:**

A total of 249 NDMM patients diagnosed between January 2008 and October 2019 were included in this retrospective study. Among them, 112 patients received vindesine-based chemotherapy (35 vDD and 77 vAD) and 137 received bortezomib-based chemotherapy (58 VDD and 79 VD).

**Results:**

In bortezomib-containing regimens, the complete response rate (48.3 *vs.* 30.4%, p = 0.033) and very good partial response or better rate (74.1 *vs*. 57.0%, p = 0.038) of VDD were significantly higher than those of VD subgroup. While no superior survival was found between VDD and VD subgroup. In vindesine-containing regimens, no statistical significance was identified between vDD and vAD in terms of response rate and survival. The occurrence rates of all cardiac AEs were similar between VDD and VD.

**Conclusions:**

The vDD regimen was similar with vAD in the aspect of response rate, survival, and toxicity in NDMM patients. The addition of PLD to VD brought deeper response without increased toxicity, while no superior survival was found.

## Introduction

Multiple myeloma (MM) is a malignant tumor that ranks second among all hematological tumors worldwide (1). It is characteristic of abnormal proliferation of bone marrow plasma cells, production of clonal immunoglobulin, and destruction of the bones (2). Chemotherapy is the main therapeutic strategy for MM. The conventional first-line chemotherapy mostly uses anthracycline containing doxorubicin, which has a certain effect and less damage to stem cells, but the side effects of conventional anthracycline are obvious (3). With the advances in cytogenetic investigations, various chemotherapy regimens based on novel drugs are emerging, which are expected to improve the prognosis of MM patients.

Pegylated liposomal doxorubicin (PLD) is a liposomal form of doxorubicin, with doxorubicin packaged in liposomes with surface-bound methoxypolyethyleneglycol in the course of pegylation (4). It has a pharmacokinetic feature characterized as longer circulation time and diminished volume of distribution to promote tumor uptake (5). On one hand, interactions between diverse circulating plasma components and the liposome surface are decreased by the hydrophilic coating of PLD formulation, thus blocking the uptake of circulating liposomes mediated by reticuloendothelial system. This allows circulating liposomes to better reach tumors which have increased vascular permeability (4). On the other hand, PLD has a particle size window of 20–200 nm, which seems to be the best opportunity to take advantage of the difference in permeability between normal and tumor vessels (6).

Clinical studies have been carried out using PLD in relapsed/refractory multiple myeloma (RRMM). PLD has been approved in combination with bortezomib for RRMM in many countries (7). In patients with RRMM, although PLD and bortezomib combination did not improve the overall survival (OS) in long-term follow-up compared to bortezomib alone (8), the results from the interim analysis showed that PLD and bortezomib significantly reduced the risk of disease progression by 45% and prolonged the median time of progression by 3 months (9). However, in Asian countries like Japan, the tolerability of dose levels which were approved in many other countries of PLD and bortezomib combination was not confirmed in RRMM patients (10). This combination treatment was prematurely discontinued in all three Japanese patients with RRMM in a phase I study due to adverse events (AEs) including Grade 3 bronchiolitis, Grade 3 peripheral sensory neuropathy, and Grade 2 stomatitis with all achieved partial response (PR). A retrospective study of 28 patients with RRMM showed PLD, bortezomib, and intravenous dexamethasone (DVD) appeared to represent a well-tolerated regimen, with only six patients (21%) showed aggravation of their baseline peripheral neuropathy (PN) and a high overall response rate (ORR) of 61%, which included one (4%) complete response (CR), three (11%) very good partial responses (VGPR), eight (29%) PR, and five (18%) minimal responses (11). In addition, DVD combination was safe and effective in elderly patients of a median age of 75 years with RRMM, with the ORR of 80% (20/25) and progression-free survival (PFS) of 8 months (12).

However, in terms of patients with newly diagnosed MM (NDMM), the antitumor efficacy and tolerability of PLD in different chemotherapy regimens have not been fully defined yet. In traditional vincristine combination regimens, compared with VAd (vincristine + doxorubicin + dexamethasone), DVd (PLD + vincristine + dexamethasone) was related to significantly less toxicity like Grade 3/4 neutropenia, a lower occurrence rate of sepsis, and less supportive care like antibiotic use, while similar efficacy, as objective response rates, PFS, and OS were similar (3). In contrary to RRMM, PLD + bortezomib therapy in a phase II study for NDMM patients did not meet the near CR/CR rate specified in the protocol, which was 7% out of 61 patients, and was associated with increased AEs in older patients (13). However, the three drug regimen VDD (bortezomib + PLD + dexamethasone) in patients with NDMM revealed well tolerance and high efficacy for induction treatment followed by HSCT in appropriate MM patients (14).

In this study, we investigated the efficacy and safety of PLD in different combination therapies based on vindesine or bortezomib in NDMM patients.

## Patients and Methods

### Patients

Patients with NDMM who received at least one cycle of chemotherapy, including PLD (PLD + vindesine + dexamethasone and PLD + bortezomib + dexamethasone) or excluding PLD (epirubicin + vindesine + dexamethasone and bortezomib + dexamethasone), between January 2008 and October 2019 in Shandong Provincial Hospital affiliated to Shandong University (SPHASU) were eligible in this retrospective analysis. The inclusion criteria were as follows: 1) newly diagnosed with symptomatic MM based on International Myeloma Working Group (IWMG) criteria (15); 2) previously untreated patients; 3) complete clinical data available for basic information as well as assessment of response and survival; 4) without clinically cardiac insufficiency (New York Heart Association Class II or greater); 5) without previous or concomitant tumor. These patients were identified through the hospital discharge registry system and electronic medical records. This study was approved by the Medical Ethical Committee of Shandong Provincial Hospital affiliated to Shandong University. All data of the recruited patients were obtained with written informed consent in accordance with the Declaration of Helsinki.

### Study Design and Treatment

This single-center, retrospective study investigated the efficacy and safety of PLD in vindesine-based regimens and bortezomib-based regimens as initial treatment for NDMM. The primary objective was CR which was assessed after every cycle of chemotherapy and before HSCT. CR was defined as: immunofixation electrophoresis (IFE) in serum and urine was negative; there was no soft tissue plasmacytoma; and the proportion of bone marrow plasmacytoma was less than 5% (16).

The chemotherapy regimens of included NDMM patients mainly consisted of vindesine-based regimens and bortezomib-based regimens, each of which was with or without PLD. The vindesine-based regimens contained vDD (PLD + vindesine + dexamethasone) and vAD (epirubicin + vindesine + dexamethasone). Patients chose vindesine-based regimens or bortezomib-based regimens mainly for economic reasons. After bortezomib entering China’s medical insurance system in 2017, most patients could use bortezomib regimens without economic pressure. The vDD regimen consisted of PLD 40 mg/m^2^ intravenously (IV) over 1 h on Day 1, as well as vindesine 1 mg/day and dexamethasone 20 mg/day orally on Days 1–4 of each 28-day cycle. The vAD regimen contained vindesine 1 mg/day and epirubicin 10 mg/day IV on Days 1–4 with dexamethasone 20 mg/day orally on Days 1–4 of each 28-day cycle. The bortezomib-based regimens contained VDD (PLD + bortezomib + dexamethasone) and VD (bortezomib + dexamethasone). The VD regimen was composed of bortezomib 1.3mg/m^2^ IV on Days 1, 4, 8, 11 and dexamethasone 20 mg/day orally on Days 1, 2, 4, 5, 8, 9, 11, 12 of every 21-day cycle. The VDD regimen consisted of the same VD regimen with PLD 40 mg/m^2^ IV on Day 1 of each 21-day cycle.

All patients’ subsequent therapies were not limited. After those who completed four to six cycles of the enrolled initial treatment, eligible patients ≤65 years without severe organ dysfunction were offered the opportunity to HSCT. HSCT was not widely used until 2016 limited by transplantation technologies and conditions. Similarly, eligible patients chose HSCT or not as for their own wishes and economic reasons. Those who refused or were not eligible for HSCT progressed to thalidomide, cyclophosphamide, lenalidomide, melphalan, ixazomib, and even cross-group therapies. Genetic abnormalities including gain (1q21), t (4;14), del (17p13) and del (13q14) were detected by fluorescence *in situ* hybridization (FISH).

### Study Assessments

#### Efficacy

We compared the treatment response and the survival time in vindesine regimens (vDD *vs*. vAD) and bortezomib regimens (VDD *vs*. VD), respectively. Response to the treatment was assessed according to the IMWG consensus criteria for response (16). The treatment response was assessed after every cycle during induction chemotherapy and before HSCT and the follow-up was conducted every 3 months during consolidation and maintenance treatment. PFS referred to the time from the beginning of treatment to disease progression or death for any cause. The definition of OS was the time from the beginning of treatment to death for any cause.

#### Safety

Safety assessment included AE monitoring, vital signs, physical examination, and clinical laboratory tests. All AEs were evaluated at each visit and graded based on the National Cancer Institute Common Terminology Criteria of Adverse Events (NCI-CTCAE), version 5.0.

### Statistical Analyses

Chi-square test or Fisher exact test was employed for categorical variables. The Kaplan–Meier method was employed to estimate the survival analysis. The log-rank test was used to calculate the PFS and OS. Statistical analyses were performed by SPSS software for Windows Version 25.0 (SPSS Inc, Chicago, IL, USA). P-value of <0.05 was considered statistically significant.

## Results

### Clinical Characteristics of Patients

**Figure 1:**
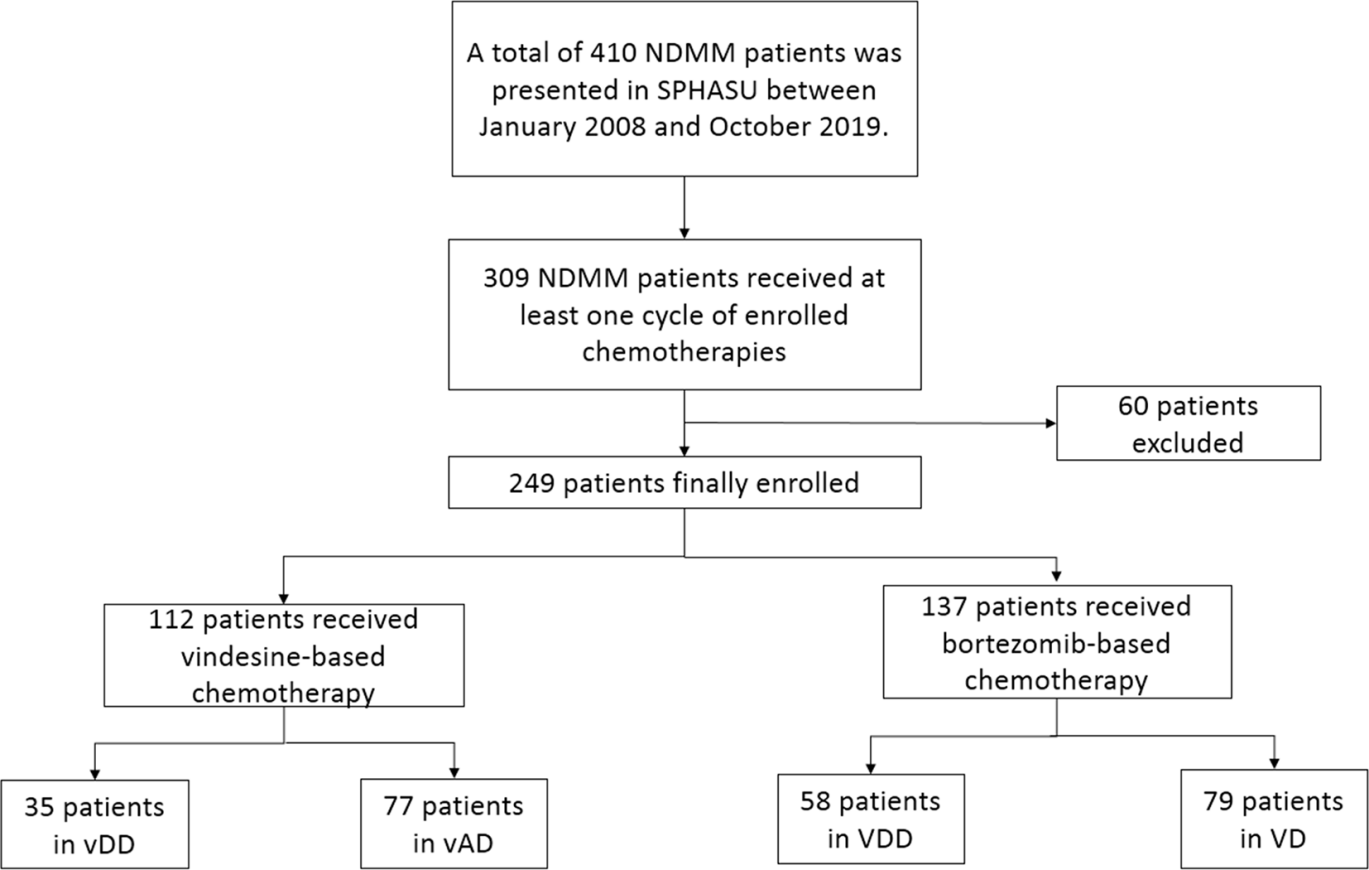
This is a flow diagram of all patients with newly diagnosed multiple myeloma enrolled and their regimen groups in this study.

A total of 410 NDMM patients was presented in SPHASU between January 2008 and October 2019. Among them, 309 NDMM patients received at least one cycle of chemotherapy, including vindesine-based regimens (vDD and vAD) and bortezomib-based regimens (VDD and VD). Based on the inclusion criteria, 249 patients were finally included in this study. We excluded patients whose clinical information data were incomplete for assessment, those with clinically cardiac insufficiency (New York Heart Association Class II or greater) and those with previous or concomitant tumor (**Figure 1**). The median age at diagnosis was 58 years for vAD, 59 years for vDD and VD, and 56 years for VDD subgroup, respectively. 46 (59.8%) patients in vAD subgroup, 27 (77.1%) in vDD subgroup, 70 (88.6%) in VD subgroup and 35 (60.3%) in VDD subgroup were male. Among all, 112 patients received vindesine-based regimens, including 35 in vDD (with PLD) and 77 in the vAD subgroup (without PLD). The median number of treatment cycles patients received was three (range, 1–11). Four patients of vDD (11.4%) and six patients of vAD subgroup (7.8%) received HSCT, respectively. 137 of the included patients were treated with bortezomib-based regimens, 58 with PLD (VDD regimen) and 79 without PLD (VD regimen). The median number of treatment cycles received was four (range, 1–8) in VDD and three (range, 1–11) in the VD subgroup. Among these two subgroups, 23 (39.7%) of the VDD subgroup and 12 (15.2%) of the VD subgroup proceeded to HSCT. The baseline clinical characteristics of patients are presented in **Table 1**.

**Table 1:** Baseline characteristics of patients.

Characteristics	vAD (n = 77)	vDD (n = 35)	P-value	VD (n = 79)	VDD (n = 58)	P-value
**Median age, years(range)**	58 (37–75)	59 (37–77)	0.272	59 (32–76)	56 (41–74)	0.218
**Gender (male)**	46 (59.8%)	27 (77.1%)	0.073	70 (88.6%)	35 (60.3%)	<0.001
**ECOG PS**						
**0–1**	65(84.4%)	31(88.6%)	0.560	72(91.1%)	52(89.7%)	0.770
**≥2**	12(15.6%)	4(11.4%)	–	7(8.9%)	6(10.3%)	–
**MM subtype**						
**IgG kappa**	23 (29.9%)	10 (28.6%)	0.889	22 (27.8%)	15 (25.9%)	0.796
**IgG lambda**	17 (22.1%)	7 (20.0%)	0.804	17 (21.5%)	12 (20.7%)	0.907
**IgA kappa**	8 (10.4%)	6 (17.1%)	0.317	7 (8.9%)	12 (20.7%)	0.048
**IgA lambda**	8 (10.4%)	4 (11.4%)	0.869	3 (3.8%)	4 (6.9%)	0.416
**IgD kappa**	0 (0.0%)	0 (0.0%)	–	1 (1.3%)	0 (0.0%)	0.390
**IgD lambda**	2 (2.6%)	0 (0.0%)	0.336	3 (3.8%)	3 (5.2%)	0.698
**Lambda light chain only**	9 (11.7%)	6 (17.1%)	0.432	13 (16.5%)	8 (13.8%)	0.669
**Kappa light chain only**	2 (2.6%)	0 (0.0%)	0.336	11 (13.9%)	3 (5.2%)	0.095
**Non-secretory**	8 (10.4%)	2 (5.7%)	0.421	2 (2.5%)	1 (1.7%)	0.750
**Genetic abnormalities**						
**Yes**	10 (13.0%)	18 (51.4%)	<0.001	28 (35.4%)	19 (32.8%)	0.744
**No**	4 (5.2%)	6 (17.1%)	0.040	22 (27.8%)	24 (41.4%)	0.098
**Not accessible**	63 (81.8%)	11 (31.4%)	<0.001	29 (36.7%)	15 (25.9%)	0.179
**ISS stage II/III**	45 (58.4%)	28 (80.0%)	0.026	70 (88.6%)	42 (72.4%)	0.015
**Durie-Salmon stage II/III**	64 (83.1%)	34 (97.1%)	0.037	70 (88.6%)	54 (93.1%)	0.375
**Median number of therapy cycles (range)**	3 (1–11)	3 (1–10)	0.369	3 (1–11)	4 (1–8)	0.422
**Subsequent transplant after treatment**	6 (7.8%)	4 (11.4%)	0.532	12 (15.2%)	23 (39.7%)	0.001

MM, multiple myeloma; ECOG PS, Eastern Cooperative Oncology Group performance status; Ig, immunoglobulin; ISS, International Staging System; Genetic abnormalities including gain (1q21), t (4;14), del (17p13), del (13q14) were detected by Fluorescence in situ hybridization (FISH).

### Response Rates

The response rates for each subgroup were summarized in **Table 2**. In the vindesine-based group, the ORR was 65.7% (23/35) in the vDD subgroup and 63.6% (49/77) in the vAD subgroup, including 17.1% (6/35) patients achieved CR, and 25.7% (9/35) patients achieved ≥VGPR in the vDD subgroup compared to 11.7% (9/77) of CR and 24.7% (19/77) of ≥VGPR in the vAD subgroup, which were all considered not statistically significant.

**Table 2:** Overall response rates of vindesine and bortezomib regimens.

	vDD (n = 35)	vAD (n = 77)	P-value	VDD (n = 58)	VD (n = 79)	P-value
**CR**	6 (17.1%)	9 (11.7%)	0.432	28 (48.3%)	24 (30.4%)	0.033
**≥VGPR**	9 (25.7%)	19 (24.7%)	0.906	43 (74.1%)	45 (57.0%)	0.038
**PR**	14 (40.0%)	30 (39.0%)	0.917	10 (17.2%)	22 (27.8%)	0.147
**ORR**	23 (65.7%)	49 (63.6%)	0.832	53 (91.4%)	67 (84.8%)	0.249
**SD**	11 (31.4%)	19 (24.7%)	0.454	3 (5.2%)	8 (10.1%)	0.292
**PD**	1 (2.9%)	9 (11.7%)	0.129	2 (3.4%)	4 (5.1%)	0.648

CR, complete response; ≥VGPR, very good partial response or better; PR, partial response; ORR, overall response rate; SD, stable disease; PD, progressive disease.

As for bortezomib-based group, although ORR between the VDD subgroup (91.4%) and VD subgroup (84.8%; p=0.249) had no significant difference, the rates of achieving CR and ≥VGPR were both significantly different between these two subgroups. 48.3% (28/58) of the patients achieved CR and 74.1% (43/58) achieved ≥VGPR in the VDD subgroup, compared to 30.4% (24/79) achieving CR (p = 0.033) and 57.0% (45/79) ≥VGPR in the VD subgroup (p = 0.038).

### Survival

**Figure 2:**
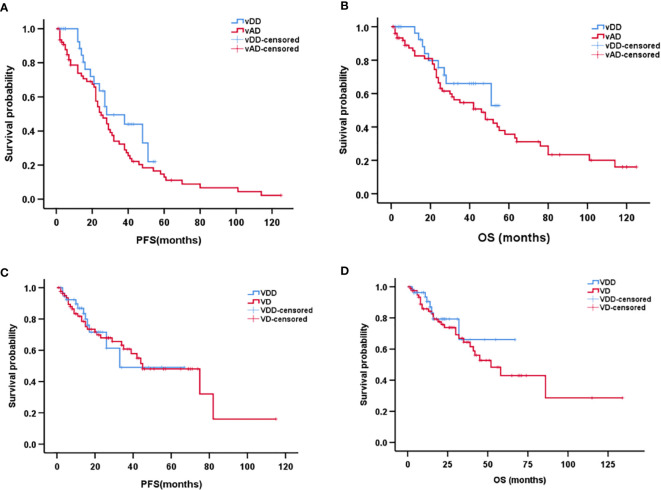
Kaplan–Meier curve of progression-free survival **(A)** and overall survival **(B)** between vDD and vAD in patients with newly diagnosed multiple myeloma. Kaplan–Meier curve of progression-free survival **(C)** and overall survival **(D)** between VDD and VD in patients with newly diagnosed multiple myeloma.

The median follow-up time for patients in the vindesine-based group was 25 months (range, 1–125 months), and the median follow-up time for patients in bortezomib-based group was 16 months (range, 1–134 months). Between the vDD and vAD subgroups, the median PFS was 28 months (95% CI, 13–42 months) in the vDD with 25 months (95% CI, 19–30 months) in the vAD. The median OS was 46 months (95% CI, 28–63 months) in the vAD while the median OS not reached in the vDD subgroup partly because of the later use of PLD. However, neither PFS nor OS differed significantly between vDD and vAD subgroup (p = 0.135, p = 0.240, respectively; **Figures 2A, B** **)**. As for bortezomib regimens, the median PFS was 45 months (95% CI, 23–66 months) and the median OS was 52 months (95% CI, 32–71months) in the VD subgroup; comparing the median PFS and the median OS both did not reach in the VDD subgroup. Similarly, no significant difference was found between the VDD and VD subgroups in either PFS (p = 0.875) or OS (p = 0.448) (**Figures 2C, D** **)**.

### Safety

In the bortezomib regimen group, with the addition of PLD, the occurrence rates of Grade 3/4 hematological toxicities, including thrombocytopenia (19.0%), neutropenia (15.5%), and anemia (5.2%), as well as infection, including pneumonia (56.9%) and urinary tract infection (5.2%), in the VDD subgroup were significantly higher than those in the VD subgroup (p = 0.004 and p = 0.005, respectively). Gastrointestinal toxicities including vomiting, diarrhea, abdominal distension, and intestinal obstruction were significantly more frequent in the VDD subgroup (46.6 *vs*. 22.8%, p = 0.003). While no treatment-related deaths occurred, and these side events can be controlled in the supportive care. The occurrence rates of all cardiac AEs were similar between the VDD and VD subgroups (p = 0.509). Among patients receiving VDD regimen, 3.4% experienced heart failure and 1.7% experienced left ventricular systolic dysfunction. The addition of PLD also did not raise the occurrence rates of herpes zoster and PN. Among all enrolled patients, only one in the VD subgroup developed pulmonary embolism.

For the vindesine-based group, the occurrence rate of infection in the vDD was significantly higher than in the vAD subgroup (51.4 *vs.* 24.7%, p = 0.005), partly because of more Grade 3/4 hematological toxicities caused by PLD. Other AEs including gastrointestinal toxicities, cardiac toxicities, herpes zoster, and PN were all without statistical significances. The results of all AEs in each group are shown in **Tables 3** and **4**.

**Table 3:** Treatment-related adverse events of bortezomib-based regimens.

Adverse events	VD (n = 79)	VDD (n = 58)	P-value
**Hematologic events (Grade 3/4)**	14 (17.7%)	23 (39.7%)	0.004
Neutropenia	2 (2.5%)	9 (15.5%)	0.006
Thrombocytopenia	8 (10.1%)	11 (19.0%)	0.139
Anemia	4 (5.1%)	3 (5.2%)	0.977
**Cardiotoxicity**	8 (10.1%)	4 (6.9%)	0.509
Arrhythmia	4 (5.1%)	0 (0.0%)	0.082
Heart failure	3 (3.8%)	2 (3.4%)	0.914
Left ventricular systolic dysfunction	0 (0.0%)	1 (1.7%)	0.241
ECG QT corrected interval prolonged	1 (1.3%)	1 (1.7%)	0.825
**Infection**	30 (38.0%)	36 (62.1%)	0.005
Pneumonia	30 (38.0%)	33 (56.9%)	0.028
Urinary tract	0 (0.0%)	3 (5.2%)	0.041
**Thromboembolism**	2 (2.5%)	0 (0.0%)	0.222
Deep vein thrombosis	1 (1.3%)	0 (0.0%)	0.390
Pulmonary embolus	1 (1.3%)	0 (0.0%)	0.390
**Gastrointestinal**	18 (22.8%)	27(46.6%)	0.003
Vomiting	1 (1.3%)	4 (6.9%)	0.082
Diarrhea	8 (10.1%)	15 (25.9%)	0.015
Abdominal distension	6 (7.6%)	4 (6.9%)	0.877
Intestinal obstruction	3 (3.8%)	4 (6.9%)	0.416
**Hepatic disorders**	4 (5.1%)	4 (6.9%)	0.651
**Skin**	21 (26.6%)	13 (22.4%)	0.577
Herpes zoster	13 (16.5%)	8 (13.8%)	0.669
Rash	8 (10.1%)	5 (8.6%)	0.766
**Peripheral neuropathy**	21 (26.6%)	16 (27.6%)	0.896

ECG, electrocardiogram.

**Table 4:** Treatment-related adverse events of vindesine-based regimens.

Adverse events	vAD (n = 77)	vDD (n = 35)	P-value
**Hematologic events (Grade 3/4)**	16 (20.8%)	12 (34.3%)	0.126
Neutropenia	11(14.3%)	11 (31.4%)	0.034
Thrombocytopenia	2 (2.6%)	1 (2.9%)	0.937
Anemia	3 (3.9%)	0 (0.0%)	0.237
**Cardiotoxicity**	8 (10.4%)	5 (14.3%)	0.551
Arrhythmia	5 (6.5%)	2 (5.7%)	0.875
Heart failure	2 (2.6%)	3 (8.6%)	0.156
Left ventricular systolic dysfunction	1 (1.3%)	0 (0.0%)	0.498
**Infection**	19 (24.7%)	18 (51.4%)	0.005
Pneumonia	16 (20.8%)	17 (48.6%)	0.003
Urinary tract	3 (3.9%)	1 (2.9%)	0.784
**Thromboembolism**	0 (0.0%)	0 (0.0%)	–
Deep vein thrombosis	0 (0.0%)	0 (0.0%)	–
Pulmonary embolus	0 (0.0%)	0 (0.0%)	–
**Gastrointestinal**	21 (27.3%)	5 (14.3%)	0.131
Vomiting	1 (1.3%)	1 (2.9%)	0.564
Diarrhea	5 (6.5%)	1 (2.9%)	0.428
Constipation	12 (15.6%)	3 (8.6%)	0.312
Intestinal obstruction	3 (3.9%)	0 (0.0%)	0.237
**Hepatic disorders**	4 (5.2%)	0 (0.0%)	0.170
**Skin**	5 (6.5%)	2 (5.7%)	0.875
Herpes zoster	1 (1.3%)	2 (5.7%)	0.180
Rash	4 (5.2%)	0 (0.0%)	0.170
**Peripheral neuropathy**	10 (13.0%)	2 (5.7%)	0.249

## Discussion

In this present study, we retrospectively analyzed the efficiency and safety of PLD in different combination therapies for patients with NDMM. In therapies based on vindesine, PLD did not show superior antitumor efficacy compared to epirubicin, with similar ORR, CR and ≥VGPR rate. Replacement of epirubicin with PLD combined with vindesine and dexamethasone did not bring about significantly longer PFS or OS.

On the other hand, the addition of PLD to bortezomib and dexamethasone demonstrated a significantly better CR rate of 48.3%, ≥VGPR rate of 74.1%, and a slightly improvement in ORR, which is in accordance with the research by Wang et al. (17), which suggested that addition of PLD resulted in a deeper remission. Preclinical studies demonstrated that the synergistic effect between bortezomib and anthracycline through the caspase-8 pathway and dexamethasone through the caspase-9 pathway provided the rationale for combining PLD to bortezomib and dexamethasone (18).

PLD is an anthracycline compound, which is used as topoisomerase II inhibitor and DNA damaging agent. The upregulation of nuclear factor-*κ*B (NF-*κ*B), which results in transcription of genes involved in oncogenes is one major mechanism that inhibits the effect of PLD. Several researches have verified that bortezomib enhances the anti-MM effects of doxorubicin by suppressing the degradation of the NF-*κ*B inhibitor to inhibit the activation of NF-*κ*B (18). Proteasome inhibitors can also stimulate the MKP-1 to anti-apoptosis. Preclinical studies suggested that anthracyclines attenuated the induction of MKP-1, thus inhibiting its antiapoptotic effect (19). Various tumor model preclinical studies showed a synergy of increased apoptotic activity in combination of doxorubicin and bortezomib, and prevented anti-apoptotic activity which both the transcription factor NF-*κ*B and the MKP-1 involved are observed (4). Thus, the combination of proteasome inhibitors bortezomib and anthracyclines PLD can increase their both efficacy (11).

Clinical data based on therapeutic approaches of MM have already indicated that the quality of response could predict long-term outcomes (20), with deeper responses, such as achievement of ≥VGPR, are associated with longer PFS and OS (14). On one hand, deeper responses of VDD did not show longer PFS or OS than the VD subgroup in our study of the total survival data. It is possible the unlimited subsequent therapy and inadequate follow-up time covered this benefit to some extent. After receiving the observed cycles of specific regimens, different patients progressed to varied therapies including HSCT, thalidomide, cyclophosphamide, lenalidomide, melphalan, ixazomib, and even cross-group therapies.

In terms of safety, PLD showed favorable hematological and non-hematological toxicity profile in clinical studies. A significantly decreased incidence of bone marrow suppression or neutropenic fever, less alopecia and decrease in cardiac function was observed in patients who received vDD regimen compared to those who received vAD (3). Treatment of vDD was significantly related to higher incidence of mostly Grades 1 and 2 hand–foot syndrome (3). Unfortunately, vDD regimen did not present any superior to vAD regimen in terms of safety in our study. Although the addition of PLD to bortezomib increased toxicity compared to bortezomib alone, mostly caused by increased myelosuppression and GI events, these toxicities were predictable and manageable through dose adjustment and supportive treatment (9). The increased non-hematological toxicities were more significant in NDMM patients aged ≥65 years (CALGB (Alliance) 10301) (13). The VDD regimen was associated with frequent grade 3/4 AEs including neutropenia, thrombocytopenia, pneumonitis and a high incidence of PN in several clinical trials (11). Yet PN was proved to be reversible (21) and dose-limiting (22). In our study, with the addition of PLD to VD, in accordance with the above studies, the incidence of Grade 3/4 hematological toxicities, infection, and gastrointestinal toxicities in VDD were significantly higher than those in the VD subgroup, which can be controlled in the supportive care like GSF and antibiotic use. Moreover, the occurrence rates of all cardiac AEs were comparable between VDD and VD subgroups, which means the addition of PLD did not raise the cardiac toxicity.

Multivariate analysis indicated that PLD could be an effective component in other regimens like bortezomib, cyclophosphamide, PLD, and dexamethasone combination (VCDD) (23, 24). Novel therapies such as the second-generation proteasome inhibitor carfilzomib, PLD, and dexamethasone (KDD), the deacetylase inhibitor vorinostat, PLD and bortezomib, the immunomodulatory drugs lenalidomide (25, 26) or pomalidomide (27) with PLD and bortezomib and so on all have high efficiency and well tolerance for RRMM patients. Especially, KDD demonstrated the ORR of 83% (20/24) and ≥VGPR rate of 54% (13/24), with the median PFS of 13.7 months (7). The vorinostat + PLD + bortezomib regimen produced an ORR of 65%, with the median PFS 13.9 months and the 3-year OS rate of 77% (28).

In this study, the regimens were the initial inductive therapies, and the sequential treatment was not limited, while VDD followed by TD (thalidomide + dexamethasone) or VTD was decided to be active in a clinical study, making more patients including those clinically defined high-risk cohort achieving maximal response before transplant (29). These better-quality responses were maintained following transplant and were related to a trend toward longer TTP and OS.

In conclusion, vDD is similar with vAD in response rate, survival, and toxicity for NDMM. The addition of PLD to VD brings deeper response without increased toxicity. However, superior survival in a long term was not proved in our study. There were still some limitations to our study, which was a retrospective one in a single center, and the amounts of recruited patients were confined. Thus, we included a mixed population of both receiving HSCT and not, which may have led to potential bias that could weaken the results. The heterogeneity in sequential regimens may have contributed to the relative variation in our findings. Moreover, because of the later use of PLD, the follow-up time of the regimens including PLD was inadequate and unequal with that of the regimens excluding PLD. Prospective randomized studies in larger population cohorts and extended follow-up time are necessary for further verifying the prognostic effect of PLD in different regimens.

## Data Availability Statement

The raw data supporting the conclusions of this article will be made available by the authors, without undue reservation.

## Ethics Statement

This study was approved by the Medical Ethical Committee of Shandong Provincial Hospital affiliated to Shandong University. All data of the recruited patients were obtained with written informed consent in accordance with the Declaration of Helsinki.

## Author Contributions

XW designed the study. YZ, DY, XG, and SH collected the clinical data. PL, XF, and YL analyzed the data. YZ wrote the paper. XZ and XW revised the paper. All authors contributed to the article and approved the submitted version.

## Funding

This work was supported by National Natural Science Foundation of China (no. 82070203, no. 81800194, no. 81770210, no. 81473486, and no. 81270598); Key Research and Development Program of Shandong Province (no. 2018CXGC1213); Shandong Provincial Natural Science Foundation (no. ZR2018BH011); Development Project of Youth Innovation Teams in Colleges and Universities of Shandong Province (no. 2020KJL006); China Postdoctoral Science Foundation (No.2020M672103); Technology Development Projects of Shandong Province (No.2017GSF18189); Translational Research Grant of NCRCH (No.2021WWB02, No.2020ZKMB01); Technology Development Project of Jinan City (No.201805065); Taishan Scholars Program of Shandong Province; Academic promotion programme of Shandong First Medical University (No. 2019QL018, No.2020RC006); and Shandong Provincial Engineering Research Center of Lymphoma.

## Conflict of Interest

The authors declare that the research was conducted in the absence of any commercial or financial relationships that could be construed as a potential conflict of interest.
